# Hydrophilized Ultrafiltration Membranes Synthesized from Acrylic Acid Grafted Polyethersulfone for Downstream Processing of Therapeutic Insulin and Cobalamin

**DOI:** 10.1007/s12010-022-03822-x

**Published:** 2022-03-31

**Authors:** N. Shiva Prasad, N. Lakshmi Gayatri, B. Naga Sandhya, S. Kalyani, Suresh K. Bhargava, Sundergopal Sridhar

**Affiliations:** 1 Membrane Separations Laboratory, Process Engineering, and Technology Transfer Division, CSIR - Indian Institute of Chemical Technology, Hyderabad, 500007 India; 2 Academy of Scientific and Innovative Research (AcSIR), Ghaziabad, Uttar Pradesh 201 002 India; 3 Royal Melbourne Institute of Technology (RMIT), Melbourne, VIC 3001 Australia

**Keywords:** Polyethersulfone, Polyethylene glycol, Acrylic acid, UV grafting, Ultrafiltration membrane

## Abstract

The present study focuses on synthesis of novel high-performance acrylic acid (AA) grafted polyethersulfone (PES) ultrafiltration (UF) membranes for purification of small therapeutic biomolecules such as urea, insulin, and cobalamin. The membranes were indigenously synthesized by adding polyethylene glycol (PEG) of 6 kDa M.Wt. as a pore former and subsequent grafting of AA using 2 to 6 wt.% concentrations under UV-induced photo grafting. Scanning electron microscopy reveals that the PEG additive profoundly influences the pore density on the membrane surface. FTIR spectra confirm the graft polymerization of AA with the PES substrate. Separation performance of the grafted membranes was evaluated to establish the trade-off between the degree of grafting and MWCO. From the experimental results, the pure water flux (PWF) of 6% grafted PES membrane was enhanced from 8.5 (PES [0] [6]) to 18.20 l m^−2^ h^−1^ (PES [6 +] [6]) in the presence of PEG pore former, respectively. The grafting concentration window of 2–6% resulted in selective membranes to altogether remove uremic toxins into the permeate with retention of high molecular size proteins. Hence, 5 and 6 wt.% AA grafted membranes exhibited > 90% rejection for insulin and cobalamin biomolecules along with 24.5 and 23.8 l m^−2^ h^−1^ bar^−1^ permeability towards urea, respectively. The process results correlate well with the MWCO values of membranes ranging from 1 to 10 kDa. This work provides the efficacy of these grafted membranes for potential application in the downstream processing of therapeutic biomolecules such as insulin and cobalamin.

**Supplementary Information:**

The online version contains supplementary material available at 10.1007/s12010-022-03822-x.

## Introduction

Biomolecule purification is an important research frontier today, as the world’s market in proteins separation and biomedical applications is growing tremendously. An ideal biomolecule purification strategy is achieved by the ease of operation and low-cost separation techniques, which have a high demand for mass production [13, 20]. In the past three decades, the utilization of membranes in the downstream processing of biomolecules has been widely practiced, such as whey protein purification [1, 33], hemodialysis [9, 30, 34, 36, 41], enzyme purification, and pharmaceutical applications. Among those, hemodialysis is one of the life-supporting filtration techniques for patients suffering from end-stage renal disease (ESRD) [15]. The process involves semipermeable membranes with different molecular weight cut-offs (MWCO) to conduct blood filtration to remove uremic toxins. Regardless of significant developments in membrane architecture and material selection, there are still unresolved problems like the trade-off between MWCO and separation efficiency [17]. Mainly, high flux hemodialysis is associated with high MWCO,as a result, patients often experience post consequences due to loss of vital proteins such as cobalamin and insulin [18]. Therefore, the design of high flux membranes with low MWCO is an urgent need to minimize the acute chronic side effects.

Ultrafiltration (UF) is one of the membrane separation processes, which is extensively used in protein concentration and hemodialysis [17]. The choice of the membrane is usually guided by its MWCO, which defines the capability of the membrane where it could reject 90% of the equivalent molecular size of the macromolecules. UF membranes having an average pore diameter in the range of 10–100 nm can be prepared from various synthetic polymers with high thermal stability, chemical resistivity, and resistance towards harsh cleaning chemicals [32, 35]. Polyethersulfone (PES) is the most widely used membrane in UF processes, especially for medical devices such as artificial organs, hemodialyzers, plasma collectors, and protein purification [15]. However, pristine PES UF membranes can invoke severe platelet and protein adhesion, causing the increased mortality of hemodialysis (HD) patients and low performance in protein purification. Several factors were involved in the platelet and protein adhesion, including morphology, hydrophilicity, molecular interactions, and surface chemistry.

Researchers have been adopted additive doping and surface modification techniques to enhance the process efficiency and biocompatibility of PES UF membranes. Otitoju et al. [22] reviewed the blending of PES with different additives such as polyvinylpyrrolidone (PVP), polyethylene oxide (PEO), and polyethylene glycol (PEG) to improve the hydrophilicity and performance of flux, solute rejection, and reduction of fouling. PEG has been extensively investigated as a hydrophilic additive and reduces protein adhesion on the membrane surface [15]. Besides, doping of PEG was also demonstrated as a pore-forming agent to enhance the surface porosity of the membranes. Rata et al. [24] prepared polysulfone (PSf) and cellulose acetate UF membranes using 1000, 2000, and 4000 Da PEG additives by phase inversion method for separation of whey protein. As per Amirilargani et al., PEG was used as a pore-forming agent to synthesize PES UF membranes by phase inversion method to study pure water flux and human serum albumin HSA rejection [2].

On the other hand, the surface modification of the membranes plays a vital role in biomolecule purification, especially for selective separation of the solute molecules through molecular sieving [24]. Taniguchi et al. [31] prepared PES membranes using six different monomers, namely [2 neutral (N-2-vinyl pyrrolidone, 2-hydroxyethyl methacrylate, 2 weak (carboxylic acids (acrylic acid, 2-acrylamidoglycolic acid, and 2 strong (sulfonic acids (3-sulfopropyl methacrylate, 2-acrylamido-2-methyl-1- propane sulfonic acid] as grafting agents. The AA modified membrane displayed excellent separation characteristics towards natural organic matter from their study. Mukherjee and Bandyopadhyaya modified the silver nanoparticles impregnated PES membrane with acrylic acid (AA to study biofouling prevention and improve water permeability [19]. Yang et al. synthesized PES UF membrane by surface grafting with different concentrations of AA to alter the degree of grafting and improve membrane structure and hydrophilicity [39]. Seman et al. studied the degree of grafting by varying the concentration of AA and N-vinylpyrrolidone monomers and duration of UV irradiation for removal of humic acid. Their study revealed that UV-grafted membranes exhibited a low tendency of humic acid fouling compared to ungrafted membranes [28]. However, UF membranes have a natural trade-off between permeation flux and selectivity towards biomolecules [5]. UV photo grafted membranes result in low MWCO due to a reduction in the pore diameter, which leads to low permeation flux. Therefore, developing UF membranes to achieve higher flux with the controlled mass transport of therapeutic biomolecules is of prime importance.

The present study aims to develop highly permeable membranes with the size-sieving ability to separate therapeutic biomolecules such as urea, cobalamin, and insulin by the UF process. The ultra-porous UF membranes were indigenously synthesized using PES polymer by adding 8% of 6 kDa PEG as an additive to improve membrane porosity. The membrane was further grafted with AA by varying the concentrations from 2 to 6% under UV-induced photo grafting. These indigenous membranes were characterized by Fourier transform infrared spectroscopy (FTIR), scanning electron microscopy (SEM), thermogravimetric analysis (TGA), and contact angle (CA) measurement to determine the structural, morphological, thermal, and hydrophilic nature of the membranes before and after modification, respectively. The membranes were tested for the degree of grafting of AA, MWCO using various molecular weights of PEG solutions, and pure water flux (PWF). The resulting membranes were applied to separate urea, cobalamin, and insulin semi-synthetic biomolecules as per the size exclusion principle. The separation specificity and improved biocompatibility of these membranes are discussed based on high permeability and retention.

## Materials and Methods

### Materials

The PES polymer was procured from Solvay, Vadodara, India, to synthesize flat sheet membranes. The solvent N-methyl-2-pyrrolidone (NMP), a crystalline compound of bismuth subnitrate (BiONO_3_) and hydrochloric acid (HCl) with 35–38% concentration, were purchased from Sd Fine Chemicals, Mumbai, India. The molecular weight cut-off of the synthesized membrane was determined using PEG with different molecular weights, i.e., 600, 1000, 2000, 4000, 6000, 10,000, and 20,000 Da, and AA were supplied from Sigma-Aldrich Chemical Private Limited, USA. A therapeutic biomolecule of urea and analytical agent *P*-dimethyl amino benzaldehyde (DMAB) were purchased from LOBA Chemie Pvt Ltd., Mumbai, India. Human mixtard insulin was procured from NOVO Nordisk India Pvt Ltd., Bengaluru, and cobalamin vials purchased from Mankind Pharma Ltd., New Delhi, India. Analytical grade purity of potassium iodide (KI) and sodium hydroxide (NaOH) was purchased from Molychem, Mumbai, India. The deionized (DI) water with TDS less than 2 ppm was used in sample preparation, and experimental studies were generated using an in-house facility cascaded reverse osmosis system.

### Methods

#### Synthesis of Flat Sheet Membranes

The UF flat sheet membranes were synthesized and analyzed for their MWCOs. The non-solvent induced phase separation (NIPS) method was used to fabricate the PES-PEG ultra-porous membranes. Initially, 17% (wt/wt) of PES in the NMP solvent was prepared to synthesize the pristine PES membrane. On the other hand, 8 wt % 6 kDa of PEG in NMP solution was prepared and stirred continuously for 30 min at 900 RPM under ambient temperature. Later, 17 wt % of the polymer was slowly added to the PEG solution and stirred for another 2 h at 45 ± 2 °C using a magnetic stirrer to form a homogeneous solution. The obtained solution was degassed and cast on polyester (PET) non-woven fabric support using a doctor’s blade. The membrane was immediately transferred into a non-solvent bath, where DI water was used as a coagulating agent. While casting the solution, the air gap between the blade and the fabric support was maintained at such a distance to obtain 60 µm of the porous layer.

#### Membrane Modification by UV-Induced Graft Polymerization

**Scheme 1:**
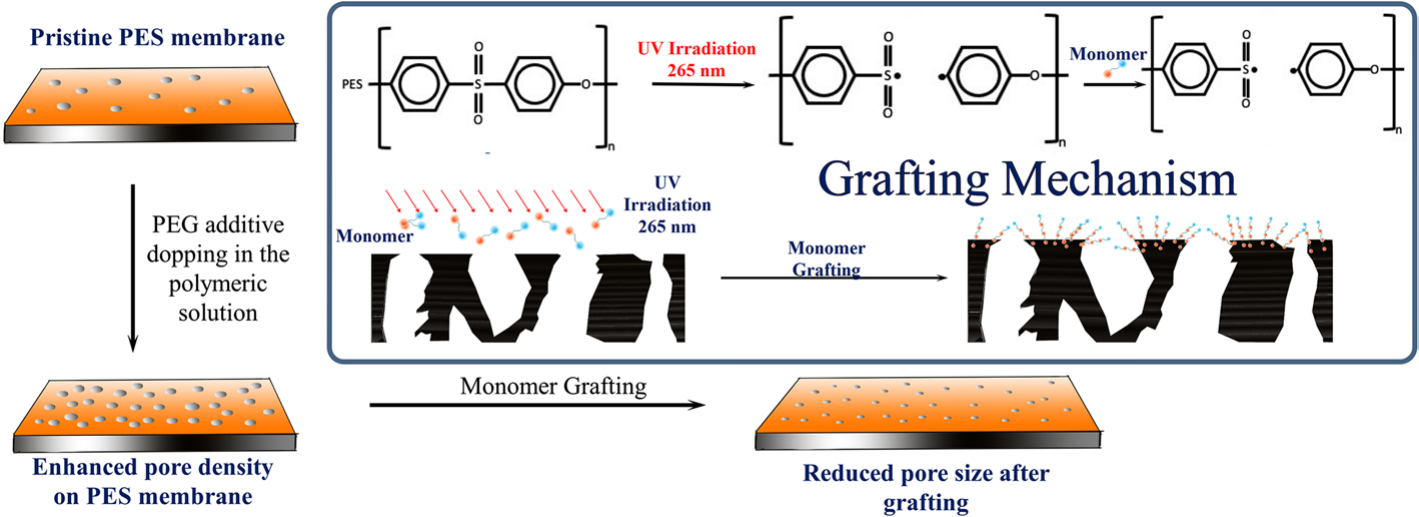
Schematic representation of AA grafting mechanism on PEG additive-loaded PES UF membrane

Weakly acidic AA was used as a monomer unit in the UV photographing of the PES membranes. Before conducting the UV irradiation, the membranes were washed thoroughly with deionized water to remove excess residual solvents from the matrix. These membranes were subjected to grafting by immersing them in an AA solution under 265 nm UV light up to 40 min. When PES substrate is exposed to UV light, it forms free radicals at the sulfate group, where a chain-growth polymerization was initiated and grafted with AA free radicals. The monomer gets attached to the PES polymer and forms polyacrylic acid (PAA) around the pores, which leads to a decrease in the membrane pore size. The reaction mechanism of UV-induced AA graft polymerization at the pores of the PES substrate was provided in Scheme 1. The grafted membranes were designated as PES[a][b], where “a” represents the molecular weight of PEG used in the UF membrane synthesis and “b” denotes the percentage of AA used for grafting, which are provided in Table 1. For example, the membrane synthesized by PES polymer solution with additive PEG 6 kDa and grafted with 6% AA represented as PES[6 +][6].

**Table 1:** Various combinations of PES membranes and their % of AA grafting

Membrane	PES Wt %	NMP Wt %	PEG Wt % (molecular weight)	AA Wt %
Pristine PES	17	83	-	-
PES[0][6]	17	83	-	6
PES[6 +][0]	17	75	8 (6 kDa)	-
PES[6 +][2]	17	75	8 (6 kDa)	2
PES[6 +][3]	17	75	8 (6 kDa)	3
PES[6 +][4]	17	75	8 (6 kDa)	4
PES[6 +][5]	17	75	8 (6 kDa)	5
PES[6 +][6]	17	75	8 (6 kDa)	6

#### Preparation of Standard Insulin, Urea, and Cobalamin (Vitamin B12) Solutions

The standard stock solution of insulin, urea, and cobalamin 1000 ppm was prepared by dissolving 1 g of insulin in 1 l of 0.01 M HCl, whereas urea and cobalamin in 1 l DI water. It was further diluted into various concentrations ranging from 20 to 100 ppm with a step of 20 ppm. The prepared standard solutions were kept aside for about 12 h to attain uniform concentrations throughout the solution [25, 40]. The solutions were employed to construct a calibration plot using an ultraviolet–visible (UV–VIS) spectrophotometer (UV-3200, LABINDIA, Maharashtra, India) at maximum wavelengths.

#### DMAB Reagent and Urea Sample Preparation for Analysis

The aldehyde-ethanol reagent solution was prepared by mixing 4 g of DMAB in 200 ml of 95% ethanol. Furthermore, 40 ml concentrated HCl solution was added to the reagent, which turned into a yellowish-green color [12]. Before analyzing the standard urea, 1 ml of the urea sample was added to 10 ml of the DMAB reagent; further makeup was done with DI water in a 25-ml volumetric flask. The obtained sample was subjected to UV–VIS spectrophotometer against a blank solution comprising 10 ml of DMAB reagent diluted with 15 ml of distilled water [21].

#### Preparation of Dragendorff and Various Concentrations of Standard PEG Solution

Dragendorff (bismuth subnitrate) reagent is widely used for quantitative analysis of PEG, which was prepared with the combination of BiONO_3_ and KI solutions. Initially, individual solutions were prepared by dissolving 1.6 of BiONO_3_ and 20 g KI in 20 ml of HCl and 50 ml DI water in brown volumetric flasks, respectively. Five milliliters of each solution was diluted with DI water in 100 ml of the brown volumetric flask [16].

Before analysis, various molecular weights of PEG standard solutions, i.e., 600, 2000, 4000, 6000, 10,000, and 20,000 Da with different concentrations of 20, 40, 60, 80, and 100 ppm, were prepared in 100-ml volumetric flasks. To determine the corresponding absorbance values of PEG, 1 ml of the prepared standard PEG was added to 8 ml of 0.01 M HCl and 1.0 ml of Dragendorff reagent.

#### UV–VIS Spectrophotometer

The UV–VIS spectrophotometer is employed for quantitative analysis of samples where light absorption is measured to estimate the concentration of solute molecules. After preparing the standard solutions, absorption spectra of each sample were analyzed against a blank solution to generate the calibration plot at their maximum wavelengths. Before calibration, the samples were scanned across the wavelength ranging from 900 to 180 nm to determine the maximum wavelength (*λ*_max_) [16]. In the present study, the maximum wavelengths for the various samples such as PEG of different MWCOs, urea, cobalamin, and insulin biomolecules were found to be 465, 420, 435, and 310 nm, respectively [21, 32, 6, 10].

#### Calibration Curve for PEG Concentration Determination

The calibration curve was generated at various known concentrations such as 20, 40, 60, 80, and 100 ppm of PEG with 1000 Da solution. Initially, the absorption spectra were recorded in the 300 to 700 nm wavelength range. From Fig. S1(a), the maximum wavelength was observed at 465 nm for PEG 1000 Da, and the absorbance values linearly increased with PEG concentration. The first-order linear regression was fitted to the recorded absorbance values at the maximum wavelength of 465 nm to establish the co-relation between PEG concentration and its corresponding absorbance, as seen in Fig. S1(b). Similarly, the calibration plots for 600, 2000, 4000, 6000, 10,000, and 20,000 Da PEG solutions were recorded using the same procedure.

### Experimental Setup

**Fig. 1:**
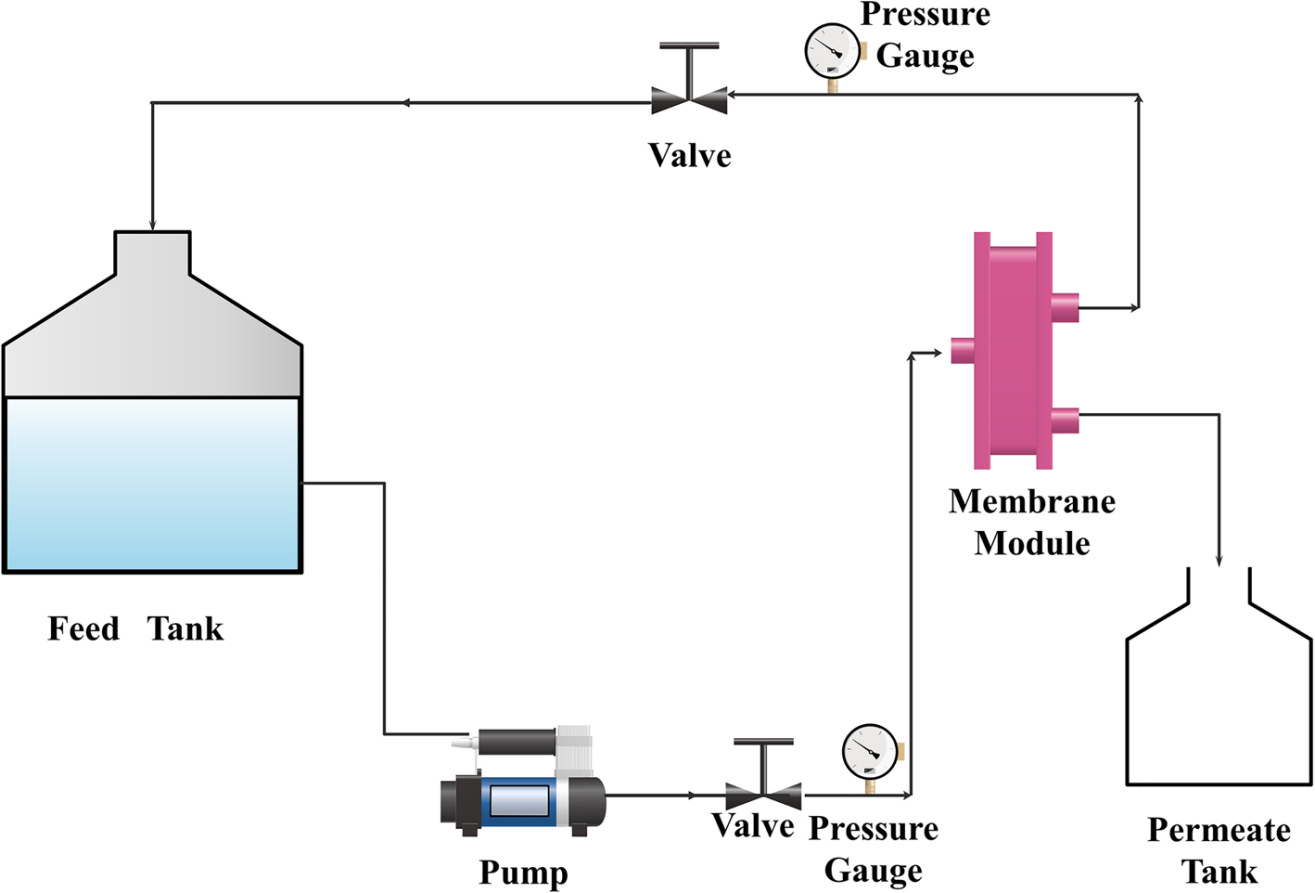
Process flow diagram of the laboratory experimental UF system

The schematic representation of the laboratory experimental setup for testing flat sheet UF membranes is shown in Fig. 1. The system consists of a 1-l capacity feed tank connected to a flat sheet membrane module having an active surface area of 90 cm^2^ through 300 gallons per day (GPD) pump. The reject stream is recycled back into the feed tank, and the volumetric flow rate of permeate stream is measured and collected for further analysis. A pressure gauge and control valves were fixed at the reject line to measure the applied pressure on the membrane module.

Initially, the feed solution was fed across the membrane under 30 psi pressure in a cross-flow configuration. Before collecting the stream samples, the permeate was recycled back to the feed for about 30 min to achieve steady-state permeation. Then, the feed and permeate samples were collected every 5 min for analysis. The UV–VIS spectrophotometer was used for quantitative analysis.

### Membrane Characterization Studies

#### FTIR

The FTIR was used to analyze the functional groups and grafting of AA on the PES surface before and after modification by a Nicolet 740, PerkinElmer 283B FTIR spectroscopy, Boston, MA, USA. The spectrum was recorded in the wavelength ranging from 650 to 4000 cm^−1^ at 25 °C.

### SEM

The surface and cross-sectional morphology of both pristine PES- and PEG-doped ungrafted membranes was analyzed using the SEM instrument with model FEI Quanta 200, USA. The grafted membranes were collected to investigate the surface morphological changes. The sample preparation step was carried out for the cross-sectional imaging, where samples were cryogenically fractured to obtain cross-sectional features. The surface SEM images were evaluated for membrane pore size distribution. Before subjecting the samples to SEM, 5 nm gold coating was provided using a sputter coater to produce a conductive layer on the surface.

#### Contact Angle (CA) Measurement

The surface hydrophilicity of pristine and PEG additive loading and various % of AA, grafted PES membranes were characterized using a sessile drop method at 25 °C. The digital microscopic instrument (model Dino-Lite Basic AM211, Taiwan) equipped with a CA analyzer was used to measure the contact angle. The angle between the baseline and gradient was measured by placing 3 μl of a water droplet on the dried membrane surface.

#### TGA Analysis

Thermal stability of the pristine and modified membranes was tested using SDT Q 600 V20.9 Build 20 analyzer, Japan. The test samples’ thermal scanning was carried out in the temperature range of 25–900 °C with a ramp heating rate of 10 °C/min under continuous N_2_ purging at 20 ml/min flow rate.

### Mathematical Equations

#### Degree of Grafting

The degree of grafting is calculated based on the amount of AA grafted on the membrane surface and can be estimated from Eq. (1);

1 $$Degree\;of\;grafting\;\left(\%\right)=\frac{W_2-W_1}{W_1}\times100$$

where *W*_1_ and *W*_2_ are the weight of the samples before and after grating the PES membrane, respectively.

#### Pure Water Flux (PWF)

The volumetric flow rate of the permeate is measured as a function of time during the UF process. The permeate flux is estimated by accounting for the volumetric flow rate per unit effective area of the membrane, as shown in Eq. (2).

2 $$\mathrm{Pure}\;\mathrm{water}\;\mathrm{flux}\;(\mathrm J)=\frac QA$$

where *Q* is the permeate volumetric flowrate and *A* is the membrane effective area*.*

#### Percentage Rejection

Percentage rejection is one of the essential parameters that define the potentiality of the membrane in particle retention. The percentage of rejection is calculated from Eq. (3).

3 $$\%\;\mathrm R\mathrm e\mathrm j\mathrm e\mathrm c\mathrm t\mathrm i\mathrm o\mathrm n=\left[1-\frac{C_p}{C_f}\right]\times100$$

where *C*_*P*_ and *C*_*F*_ are the solute concentrations of permeate and feed, respectively.

## Results and Discussion

### Study on Membrane Morphology by SEM

**Fig. 2:**
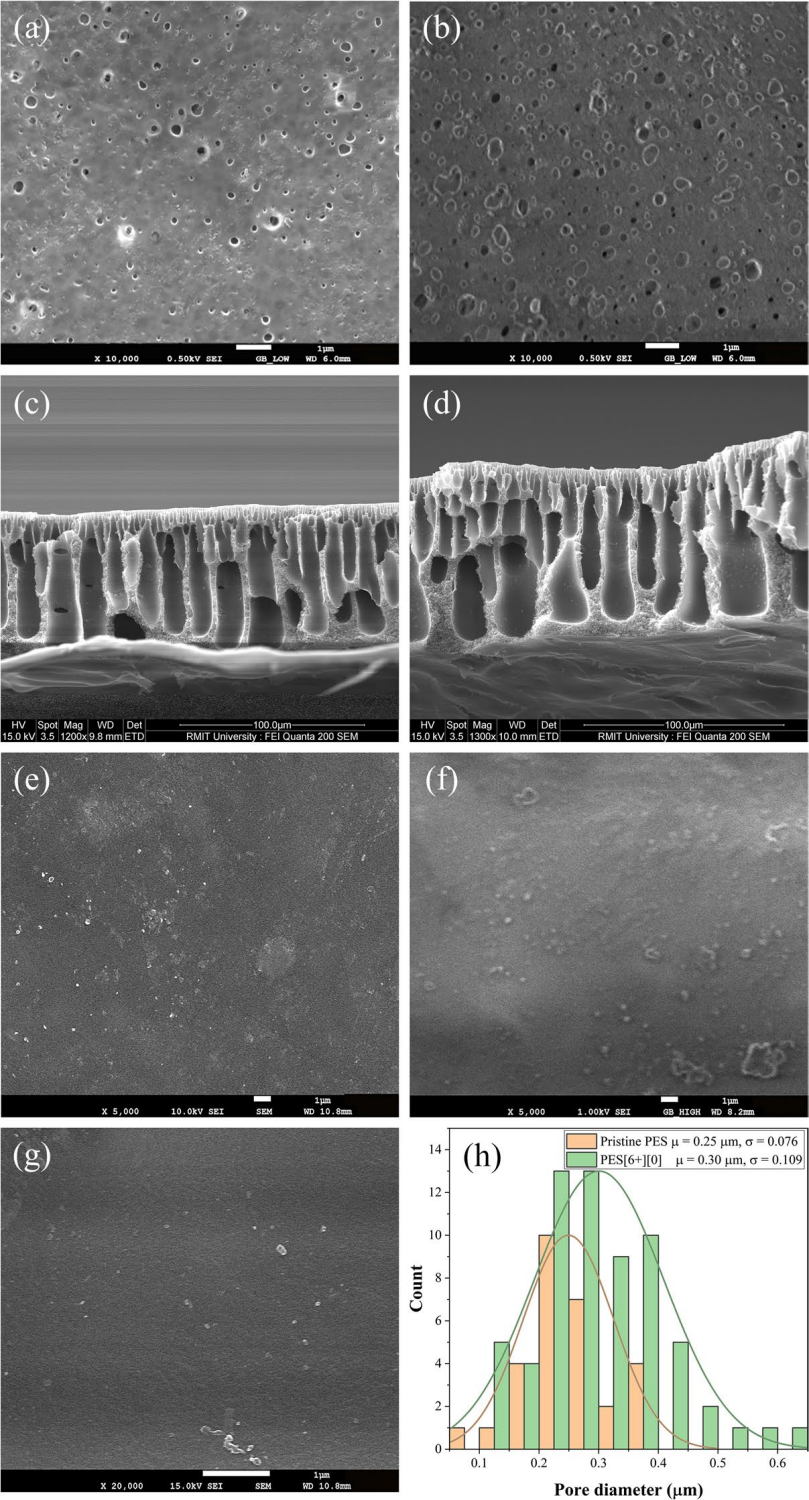
Surface morphologies of **a** pristine PES, **b** PES[6 +][0], cross-sectional morphology of **c** pristine PES and **d** PES[6 +][0], surface morphologies of grafted **e** PES[6 +][2], **f** PES[6 +][4], and **g** PES[6 +][6] membranes, **h** pore size distribution of pristine PES and PES[6 +][0]

Morphological images of the pristine PES, PES[6 +][0], and surface grafted membranes are shown in Fig. 2. The surface morphology of pristine PES and PES[6 +][0] depicted the influence of PEG additive on the pore size distribution. From Fig. 2a and b, it can be clearly evident that the surface pore density of PES[6 +][0] was enhanced in the presence of a PEG additive. The pore size distribution of the pristine PES and PES[6 +][0] was determined using an open-source image analysis tool (ImageJ, https://imagej.nih.gov). The details of the image analysis procedure are similar to that of Shahruddin et al. [29]. The pore size distribution represents the population analysis of various pore diameters present on the membranes’ surface. The statistical analysis presented in Fig. 2h indicates the mean pore diameter of pristine PES enhanced from 0.25 to 0.30 µm when PEG additive was added (PES[6 +][0]). Additionally, the pore diameters’ frequency suggests that the pore population also enhanced. However, the cross-sectional morphology of the base membranes (pristine PES and PES[6 +][0]) presented in Fig. 2c and d shows similar morphology of finger-type macrovoids consisting of a compact top layer. The formation of asymmetric cross-sectional morphology by the NIPS method was previously discussed by Wienk et al. [37].

On the other hand, the surface morphology of PES[6 +][0] grafted by various percentages of AA, i.e., 2, 4, and 6%, is shown in Fig. 2e, f, and g, respectively. Surface pore shrinking and pore blocking by crosslinking of AA monomer are clearly evident from Fig. 2e–g. The surface morphological of base and AA grafted membranes revealed significant changes in the topography, where no visible pores have been observed after grafting [42]. This observation supports the formation of the PAA layer on the porous substrate. From the morphological findings, the pore density initially increased with the PEG additive. The various AA graftings helped alter the pore diameter further, which helped develop membranes of different MWCO.

### FTIR Studies

**Fig. 3:**
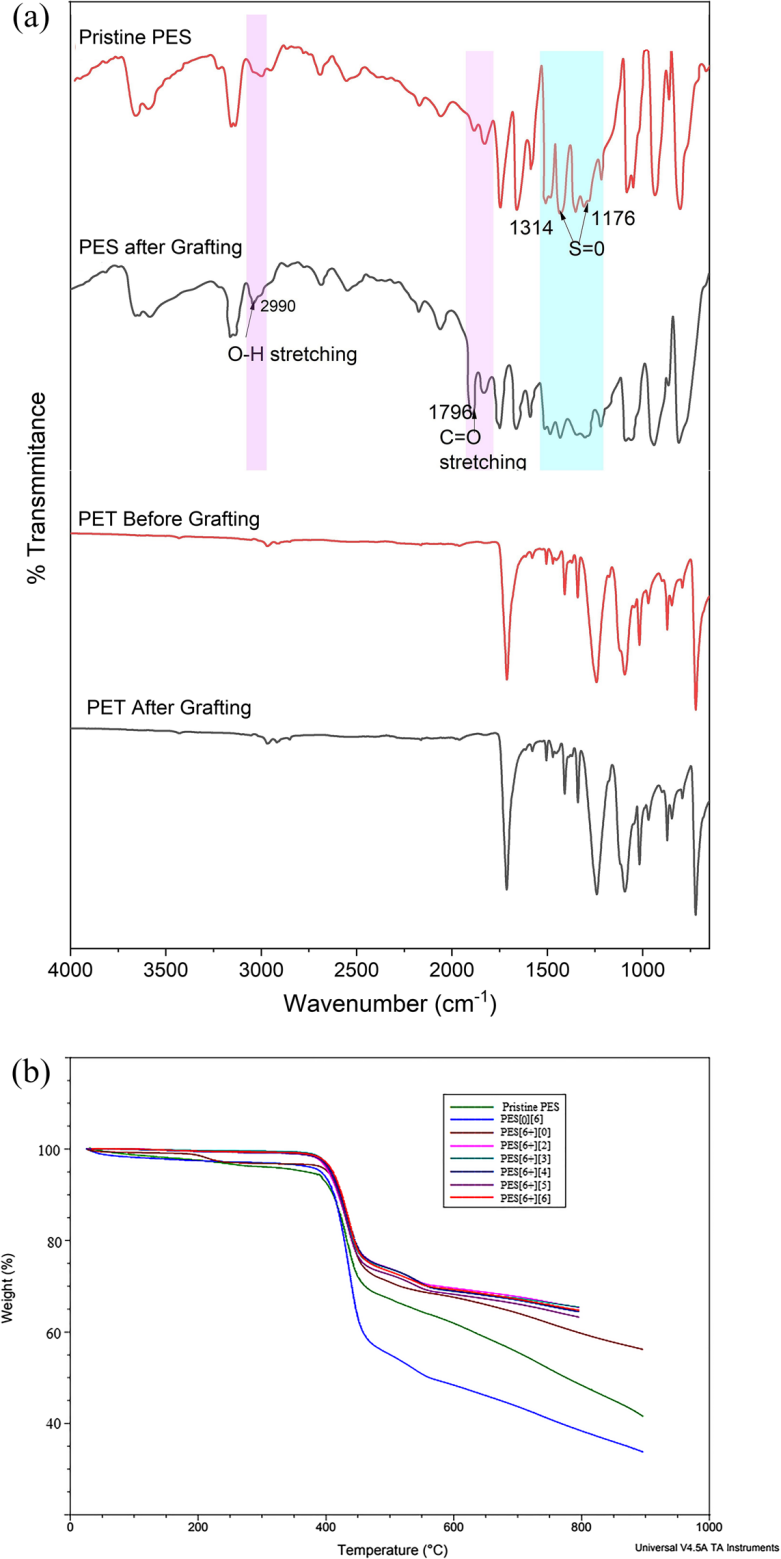
FTIR spectra of (a) pristine PES, (b) grafted PES, (c) PET before grafting, and (d) PET after grafting. **b** TGA of pristine, PES[6][0], PES[0][6], and PES[6 +][2] to PES[6 +][6] membranes

The FTIR spectra of the pristine PES and surface grafted with AA functional groups are presented in Fig. 3a. The pristine PES membrane exhibited peaks at 1176 and 1314 cm^−1^ representing the symmetric and asymmetric stretching vibration of O = S = O groups. The peaks at 1437, 1484, and 1576 cm^−1^ were attributed to the aromatic ring C = C stretching vibrations. The ether (C–O–C) group stretching and bending vibrations were found to be around 1009 cm^−1^ and 1239 cm^−1^, respectively [4]. These functional group characteristic peaks of functional groups that appeared in Fig. 3a confirm the structure of the PES membrane. The surface modification of PES with AA using graft polymerization is confirmed by identifying the new peaks at 1796, and 2990 cm^−1^ represents the carbonyl (C = O) and hydroxy (-OH) groups stretching vibrations of AA. Similar spectral patterns previously were reported by Homayoonfal et al. [14], which confirms the structural modification of the membrane. The characteristic peaks in the range of 1100 to 1400 cm^−1^ shift with the lower intensity from pristine to grafted PES membrane, confirming the possible bond formation between the sulfonated functional groups of PES with AA groups [4, 6]. The crosslinking mechanism at the pores provided in Scheme 1 was confirmed from FTIR.

On the other hand, the FTIR spectra of the polyester fabric support before and after grafting are shown in Fig. 3a. The fabric structure consists of acidic anhydrides, esters, and aromatic rings with stretching and bending vibrations. The carbonyl stretching vibration is attributed at 1710 cm^−1^, whereas the peaks in the range of 1236 to 1096 cm^−1^ represent stretching and bending vibrations of anhydride. The aromatic ring C = C and C-H stretching vibrations can be observed at 1405 and 2961 cm^−1^, respectively [7]. Figure 3a shows that the functional groups of the PET are not affected during photo grafting, which confirms AA grafting occurred at the location where the UV light exposed the PES surface sites.

### Membrane Hydrophilicity

Contact angle (CA) measurements were performed to assess the hydrophilicity of the prepared membranes. A higher degree of CA indicates the hydrophobic surface, whereas reduction in CA represents the membrane’s nature, which gradually shifts towards hydrophilic [38]. The contact angle of the pristine PES, PES[6 +][0] and various concentrations of AA grafted (PES[6 +][2]—PES[6 +][6]) membranes were measured (Table 2), among which PES-loaded PEG membrane (PES[6** +**][0]) exhibited a contact angle of 55.7° lower than pristine PES, which is due to the presence of encapsulated PEG in the membrane matrix [11]. On the other hand, the grafted PES in the absence of PEG (PES[0][6]), the membrane contact angle was found to be 60.3°, which is due to the free carboxylic acid groups of AA on the membrane surface. The contact angle further decreased from 53.2 to 41.4° with the increase in the degree of grafting in PES[6 +][2] to PES[6 +][6], respectively. These observations indicate that the graft polymerized PEG-doped base membranes became more hydrophilic, intuitively enhancing the permeation ability [39].

**Table 2:** Contact angle and degree of grafting of the pristine and AA grafted PES membranes

S. No	Membrane name	Contact angle in degrees	% grafting
1	Pristine PES	65.4	-
2	PES [0][6]	60.3	17.2
3	PES [6** +**][0]	55.7	-
4	PES [6** +**][2]	53.2	12.4
5	PES[6** +**][3]	50.5	13.6
6	PES[6** +**][4]	44.2	14.3
7	PES[6** +**][5]	42	15.5
8	PES[6** +**][6]	41.4	17.8

### Effect of AA Concentration on the Degree of Grafting

The AA concentration in the grafting bath was varied from 2 to 6% to understand the trade-off. All the membranes were grafted under the same operational condition, i.e., UV light working distance and grafting period. From Table 2, it can be observed that the grafting percentage increased from 12.4 to 17.8%, with an increase in the percentage of AA monomer. The degree of grafting is enhanced on the membrane surface due to abundantly available monomer units at higher concentrations.

The thermographs of pristine PES, PEG-loaded PES (PES[6 +][0]), without PEG-loaded AA grafted PES (PES[0][6]) and various % of AA grafted membranes, i.e., PES[6 +][2] to PES[6 +][6] is illustrated in Fig. 3b. The pristine PES- and PEG-loaded PES[6 +][0] membranes exhibited the single-stage weight loss from 400 to 420 °C, whereas the grafted membranes, i.e., PES[0][6], PES[6 +][2] to PES[6 +][6], exhibited an additional weight loss from 450 to 550 °C, which corresponds to the decomposition of AA. From Fig. 3b, it can be clearly evident that the second derivative weight loss increased with the increasing graft solution concentration, which confirms the degradation of PAA [39]. From TGA analysis, it can be concluded that the PAA deposition on the PES porous substrate is consistent with the degree of grafting.

### MWCOs of PES Membranes and Pure Water Flux (PWF)

**Fig. 4:**
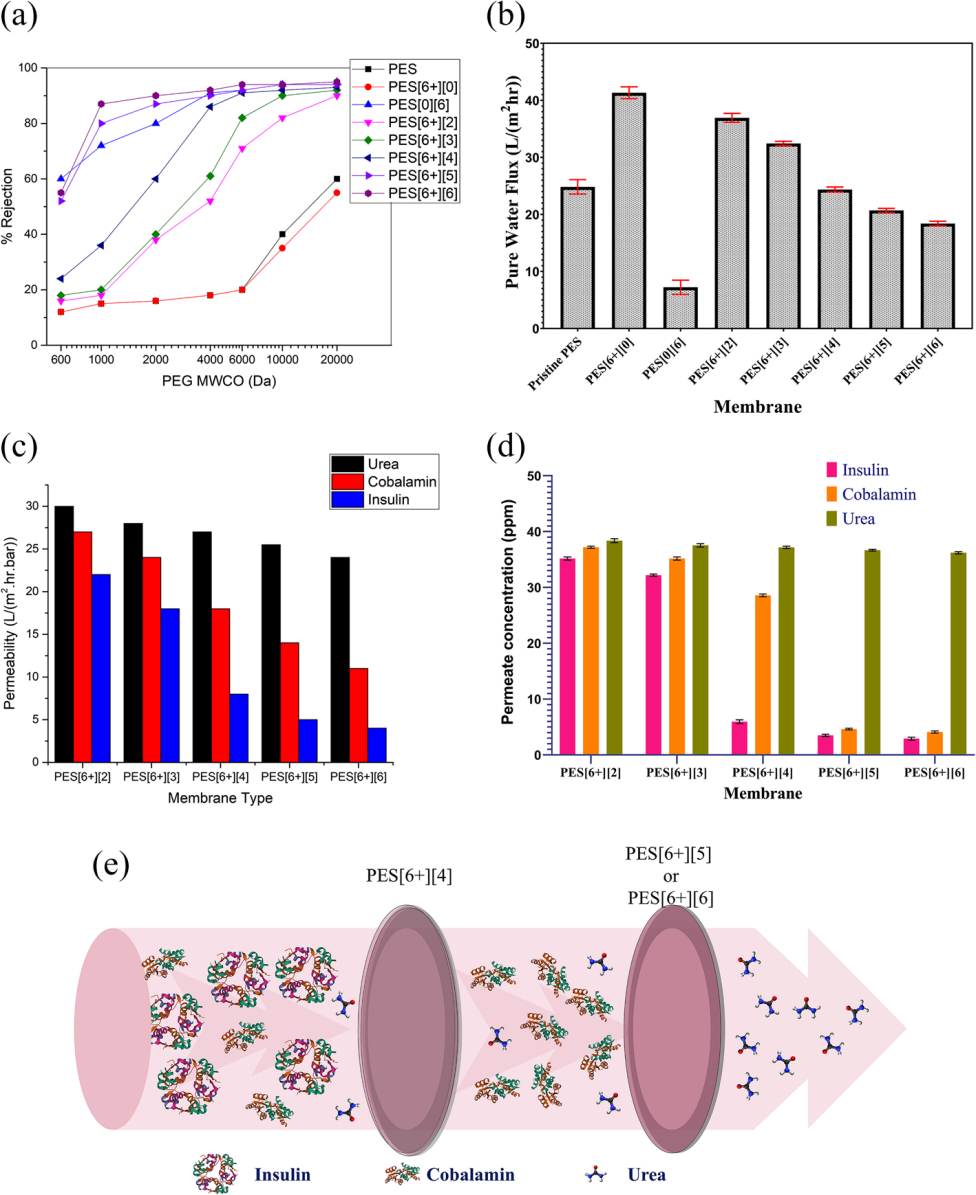
Effect of grafting on MWCOs of the membrane, **b** effect of degree of grafting on PWF, **c** permeability trend of urea, cobalamin, and insulin for PES[6 +][2] to PES[6 +][6] membranes, **d** effect membrane grafting on permeate concentration, **e** schematic of transport phenomenon of biomolecules

The influence of the degree of grafting (AA) on the performance of the PES membranes was investigated using molecular weight cut-off (MWCO) and PWF. MWCO evaluation is a pivotal characterization technique in porous films to demonstrate the retention capabilities of the membrane, which represents > 90% rejection of solute molecules. In the present study, PEG with different molecular weights, i.e., 600, 1000, 2000, 4000, 6000, 10,000, and 20,000 Da, were tested for their retention through the grafted membranes, and the corresponding % rejections are graphically illustrated in Fig. 4a. The rejection of each PEG solution gradually increased with the degree of grafting on the membrane surface due to greater water flux, a consequence enhanced hydrophilic nature of the membrane. The base membranes (pristine and PES[6 +][0]) exhibited 60% rejection for 20 kDa, which implies the MWCO of these membranes is more than 20 kDa. From Fig. 4a, the pristine and additive doped membranes without grafting (PES[6 +][0]) exhibited a similar rejection pattern. However, after doping PEG as an additive, the membrane PWF (Fig. 4b) was enhanced from 25.03 to 41.50 L m^−2^ h^−1^, which is possibly due to increased pore density on the membrane surface [27]. On the other hand, PES grafted (PES[0][6]) without PEG doping membrane showed 89% rejection towards 4 kDa PEG aqueous solution, whereas the PWF drastically reduced from 25.03 to 8.5 L m^−2^ h^−1^ (Fig. 4b). This phenomenon is due to the UV photo grafting on the pores of the base membrane which are shrinking or blocking with the PAA layer [14]. Therefore, the MWCO of grafted pristine (PES[0][6]) membrane decreased from 20 to 4 kDa with a reduction in the PWF. However, the reduction in PWF indicates the increase in the hydraulic resistance for the permeation, which is undesirable in the design of high-performance MWCO membranes. From the above findings, it can be concluded that the PEG additive enhances the PWF by increasing the pore density, which SEM supports. Variably, the grafting on the membrane surface could fine-tune the MWCO of the membrane for the effective retention of solute molecules [8]. Thus, combining additive doping followed by various degrees of grafting makes it possible to synthesize high-performance MWCO membranes [23].

Moreover, it can also be observed from Fig. 4a that the % rejection of various AA grafted membranes, i.e., PES[6 +][2] to PES[6 +][6], exhibited different MWCOs. The PES[6 +][2] membrane allows all the PEG solutions ranging from 0.6 to 20 kDa, which indicates the membrane has MWCO more than 20 kDa. However, the % rejection for 10 and 20 kDa significantly improved with the 2% AA grafting (PES[6 +][2]) compared to base membranes, which is due to the pore grafting on the surface. In PES[6 +][3], the membrane permeated the PEG solutions up to 10 kDa and retained the 20 kDa, reflecting the membrane MWCO between 10 and 20 kDa. Four percent AA grafted membrane PES[6 +][4] achieved 90% rejection for 6 kDa PEG solution, which indicates the MWCO of the membrane was altered to 6 kDa. Furthermore, the resulting PES[6 +][5] and PES[6 +][6] membranes offered 87 and 90% rejections for 2 kDa PEG solution, which confirms the MWCO can be approximately 2 kDa, respectively. Hence, this infers that the MWCO is less than 2 kDa for PES[6 +][5] and PES[6 +][6] membranes, which have negligible variation in pore size [43]. On the other hand, from Fig. 4b, it can also be observed that the PWF gradually decreased from 38.5 to 18.2 for PES[6 +][2] to PES[6 +][6] membranes, respectively, which is consistent with MWCO of the present study. From the preceding observations and results, it can be inferred that the pore density has increased with the additive, which led to enhancement in the PWF—followed by the degree of grafting which influenced the MWCO of the membranes, with the conjugate effect propagating to high-performance for selective separation.

### Application of PES[6 +][2] to PES[6 +][6] Membranes for Separation of Therapeutic Biomolecules

UV-induced photo grafting of PAA on the PES membranes demonstrated a wide array of MWCO membranes with enhanced permeability by varying the graft solution concentration and additive doping [14]. To examine the separation characteristics of the AA grafted membranes, downstream processing of therapeutic biomolecules such as urea, cobalamin, and insulin was carried out. Fig. 4c represents the permeability data of urea, cobalamin, and insulin solutions of 50 ppm concentration at various AA grafted PES from (PES[6 +][2] to PES[6 +][6]) membranes. From the graphical observation, it can be inferred that the permeability was decreased from 30 to 23.8 L h^−1^ m^−2^ bar^−1^ for urea from PES[6 +][2] to PES[6 +][6], respectively. Similarly, the permeability of cobalamin and insulin was decreased from 27 to 12 and from 22 to 4 L h^−1^ m^−2^ bar^−1^, respectively (Fig. 4c). The decreasing permeability trend is due to the molecular sieving of biomolecules in the order of urea < cobalamin < insulin. As per Zhu et al., the permeation rates of grafted membranes follow the molecular sieving mechanism for small molecules [43].

From Fig. 4d, it can be seen that a negligible declination in permeate concentration of urea was observed for PES[6 +][2] to PES[6 +][6] membranes at 30 psi pressure. The reason behind this is due to the smaller molecular size of the urea, i.e., < 0.5 kDa, allowed by these membranes. On the other hand, the permeability of cobalamin and insulin solutions followed a similar trend to PES[6 +][2] and PES[6 +][3]. However, in the case of the membrane PES[6 +][4], a drastic declination can be observed in the insulin concentration in the permeate stream, whereas negligible change was observed in the case of cobalamin. Moreover, the PES[6 +][5] and PES[6 +][6] membranes (Fig. 4d) exhibited retention of insulin and cobalamin, and the concentration of these molecules was found to be minimal in the permeate. Therefore, the insulin and cobalamin permeability and permeate concentrations decreased with increasing the degree of grafting on the membranes.

Additionally, the membrane’s molecular sieving and size exclusion phenomena help to allow the low molecular weight biomolecule (urea) to permeate through the membrane faster than other therapeutic molecules. The size exclusion of biomolecules is evaluated in terms of the membrane transport mechanism provided in Fig. 4e. From the graphical and transport properties of the grafted membrane, it can be concluded that these membranes can pass urea molecules easily and concentrate the remaining biomolecules at the reject stream. Hence, from the overall characterization and experimental observations, it can be confirmed that these indigenously synthesized flat sheet grafted membranes demonstrated excellent permeability for the semi-synthetic biomolecules of the present study and are useful for separation of these therapeutic molecules with MWCO range from 1 to 10 kDa. Appropriate selection of grafting agent and optimized degree of grafting can result in membranes suitable for downstream processing and separation of biomolecules.

## Conclusions

The PES UF membranes were indigenously synthesized by doping 6 kDa PEG followed by various degrees of AA grafting on the membrane surface through UV-induced photo-catalytic reaction. The pore former of PEG enhanced the surface porosity, and AA grafting helped to control the pore diameter and MWCO of the membrane. The FTIR spectra confirmed the formation of new bonds between AA with the sulfone groups of the PES. The thermal analysis of the grafted membranes exhibited an additional weight loss of PAA, which supports the structural analysis. The trade-off between the grafting solution concentration with a degree of grafting and corresponding MWCOs of the prepared membranes was successfully established. From the experimental results, the PWF of the grafted membranes was significantly enhanced. The PES[6 +][4] membrane permeated cobalamin and retained the insulin biomolecules, whereas PES[6 +][5] and PES[6 +][6] membranes completely retained insulin along with cobalamin. The low molecular weight urea biomolecule permeated through both grafted and pristine membranes. Hence, the essential peptides and proteins present in human blood above 2 kDa will be retained, whereas these membranes can remove the uremic toxins of molecular weight less than 1 kDa. Therefore, from the experimental results, it is worth mentioning that PES[6 +][6] and PES[6 +][5] membranes can be used to remove uremic toxins for renal patients instead of tedious dialysis procedures by surgically implanting this membrane cassette into the chronic renal patients. When the permeate is attached to the urinary bladder and the reject is sent back to the bloodstream, the uremic toxins can be simultaneously removed through this effective process. Thus, through the UF process, grafted membranes can continuously remove uremic toxins to reduce the urea-peptide hybridization in the bloodstream.

## Supplementary Information

Below is the link to the electronic supplementary material.

Supplementary file1 (DOCX 199 KB)

## Data Availability

Not applicable.
