# Supplementary material for: Hydrophilized Ultrafiltration Membranes Synthesized from Acrylic Acid Grafted Polyethersulfone for Downstream Processing of Therapeutic Insulin and Cobalamin
Source: Appl Biochem Biotechnol. 2022 Mar 31;194(8):3400–18. doi: 10.1007/s12010-022-03822-x (PMC9270308; doi:10.1007/s12010-022-03822-x)
Supplement: Supplementary file 1 — Supplementary file1 (DOCX 199 KB) [file 12010_2022_3822_MOESM1_ESM.docx]

**Hydrophilised Ultrafiltration Membranes Synthesized from Acrylic Acid Grafted Polyethersulfone for Downstream Processing of Therapeutic Insulin and Cobalamin**

N. Shiva Prasad^1,2,3^, N. Lakshmi Gayatri^1^, B. Naga Sandhya^1^, S. Kalyani^1^,

Suresh K Bhargava^3^, Sundergopal Sridhar^1,2*^

*^1^ Membrane Separations Laboratory, Process Engineering, and Technology Transfer Division, CSIR - Indian Institute of Chemical Technology, Hyderabad, India-500007.*

*^2^Academy of Scientific and Innovative Research (AcSIR),* *Ghaziabad, Uttar Pradesh 201 002, India*

*^3^ Royal Melbourne Institute of Technology (RMIT), Melbourne, VIC 3001, Australia*

* Corresponding author; E-mail ID: sridhar11in@yahoo.com, Tel: +91-040-27193408

**Fig. S1 (a) Absorption spectra of various concentrations of PEG 1,000 kDa solution and (b) The calibration curve of PEG 1,000 kDa**


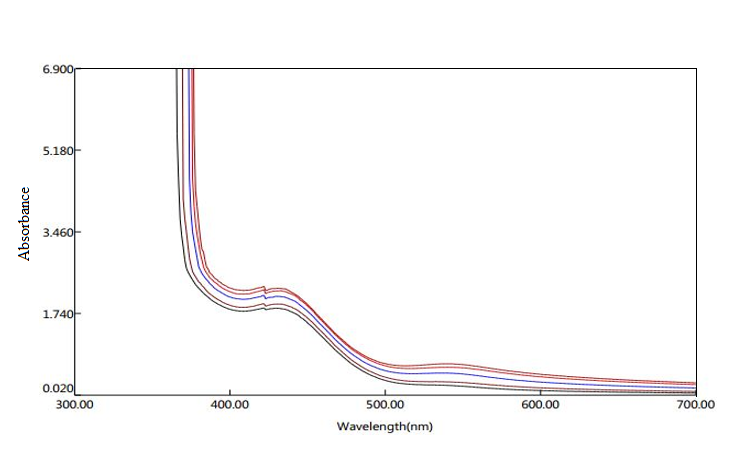


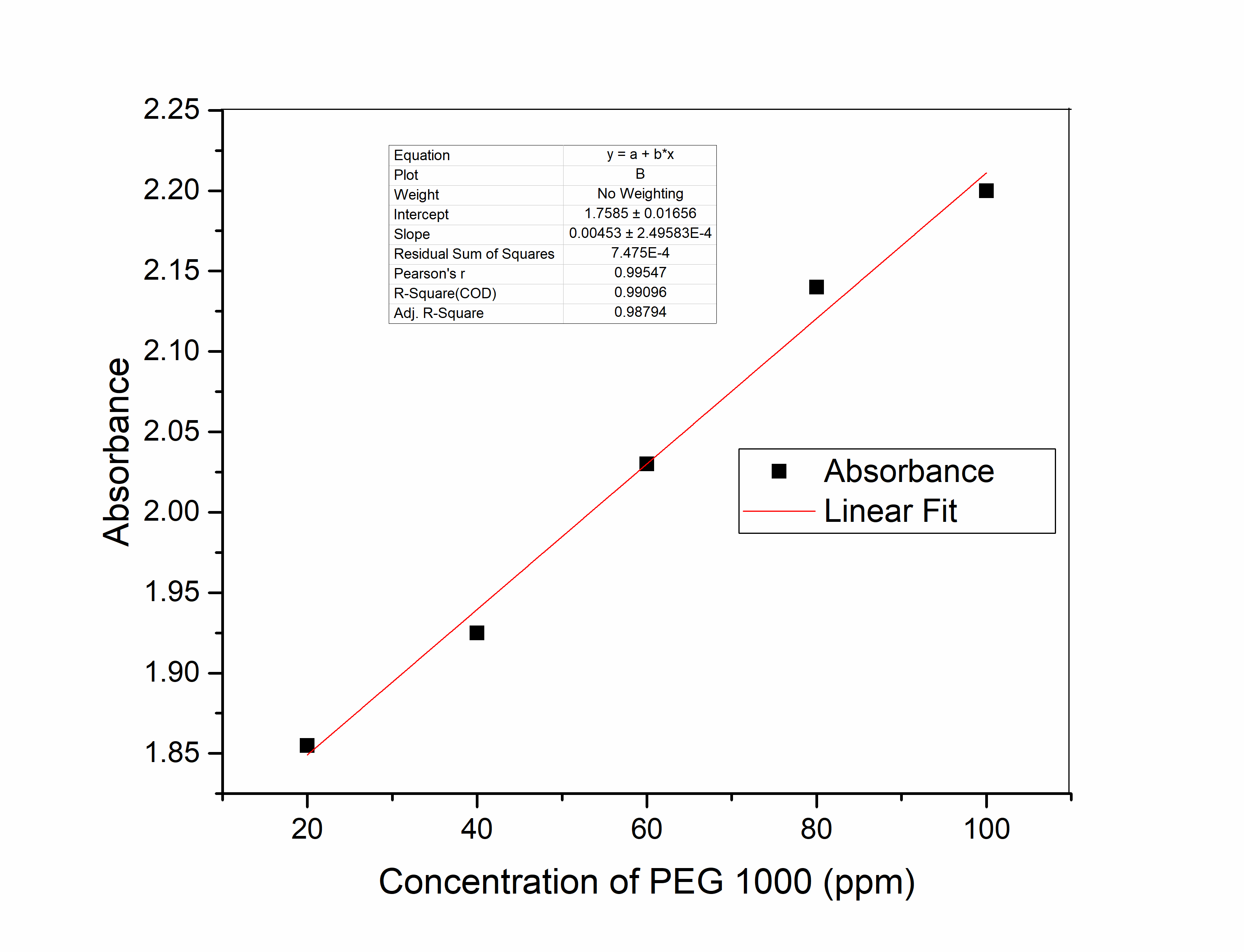


(b)

**Fig. S1**
